# Neoadjuvant immunotherapy for nonmetastatic dMMR/MSI colon cancer: a real-world retrospective AGEO study

**DOI:** 10.1016/j.esmoop.2025.105516

**Published:** 2025-07-31

**Authors:** C. Lemaire, A. Boileve, G. Manceau, C. Coutzac, M. Muller, P. Girot, L. Lellouche, A. Saltel-Fulero, C. Lagorce-Pages, C. Gallois, A. Gandini, M. Karoui, J. Taieb, J. Palle

**Affiliations:** 1 Université Paris Cité, Department of Digestive Oncology, CARPEM Comprehensive Cancer Center, Hôpital Européen Georges Pompidou, Assistance Publique—Hôpitaux de Paris, Paris, France; 2 Department of Medical Oncology, Gustave Roussy, INSERM U1279, Villejuif, France; 3 Université Paris Saclay, Orsay, France; 4 Université Paris Cité, Department of Digestive Surgery, CARPEM Comprehensive Cancer Center, Hôpital Européen Georges Pompidou, Assistance Publique—Hôpitaux de Paris, Paris, France; 5 Department of Oncology, Centre Léon Bérard, Lyon, France; 6 Department of Gastroenterology and Digestive Oncology, Université de Lorraine, CHRU de Nancy, Vandoeuvre-lès-Nancy, France; 7 Department of Gastroenterology and Digestive Oncology, Centre Hospitalier Loire Vendée Océan, La Roche sur Yon, France; 8 Université Paris Cité, Department of Gastroenterology and Digestive Oncology, Hôpital Cochin, Assistance Publique—Hôpitaux de Paris, Paris, France; 9 Université Paris Cité, Department of Radiology, CARPEM Comprehensive Cancer Center, Hôpital Européen Georges Pompidou, Assistance Publique—Hôpitaux de Paris, Paris, France; 10 Université Paris Cité, Department of Digestive Anatomopatology, CARPEM Comprehensive Cancer Center, Hôpital Européen Georges Pompidou, Assistance Publique—Hôpitaux de Paris, Paris, France

**Keywords:** immune checkpoint inhibitors, neoadjuvant immunotherapy, dMMR/MSI colon cancer, nonmetastatic, microsatellite instability, signet ring cell

## Abstract

**Background:**

Several studies showed that in mismatch repair-deficient (dMMR)/microsatellite instability (MSI) nonmetastatic colon cancer (CC), neoadjuvant immune checkpoint inhibitors (ICIs) were associated with exceptional rates of pathological major response (pMR) and complete response (pCR). Patients included in these trials, however, were highly selected, and real-life data are now needed to better evaluate the efficacy and tolerability of neoadjuvant ICIs in routine clinical practice.

**Patients and methods:**

This retrospective observational study aimed to describe the clinical practices regarding ICIs in the neoadjuvant setting for patients with dMMR/MSI nonmetastatic CC, and to evaluate their efficacy and safety in real-world conditions. Patients receiving ICIs as part of a therapeutic trial were excluded.

**Results:**

Between 2019 and 2024, 32 patients were included across six French centers [median age 70 years (range 51-76 years), Lynch syndrome 31%]. Twenty-four patients had right-sided CC (85%), including three (9%) with two primary tumor locations. Ten patients (31%) received ipilimumab + nivolumab (NICHE regimen), while 22 (69%) were treated with pembrolizumab monotherapy. Grade ≥3 toxicities were observed in six patients (19%), including one toxic death and three toxicities (9%) leading to treatment discontinuation (one rheumatoid polyarthritis-like syndrome and two tumor fistulizations). Three patients developed bowel obstruction while receiving ICIs, two of whom underwent surgery showing pCR and pMR. Thirty patients were resected and 33 tumors were analyzed histologically with pMR in 21 cases (64%) including pCR in 14 cases (42%). The presence of an independent-cells contingent was statistically associated with poor pathological response.

**Conclusions:**

This retrospective real-world study confirms the excellent results of neoadjuvant ICIs in dMMR/MSI nonmetastatic CC patients. pMR and pCR rates were, however, lower than those published in previous studies (64% and 42%, respectively), with higher rates of grade ≥3 toxicity, including one potential toxic death and three treatment discontinuations.

## Highlights

• In phase II studies, neoadjuvant ICI induce exceptional response rates in MSI locally advanced colon cancer.
• In real world-patients, pathological response rates seem, however, below those reported in clinical trials.
• This observational study showed lower major pathological response and pCR than those reported and more sever toxicities, including one suspected toxic death.
• More standardized patient selection criteria and treatment regimen are needed to optimize tolerability and efficacy.

## Introduction

Colorectal cancer (CRC) affects 1.9 million new patients annually and caused 904 000 deaths worldwide in 2022.^1^ Microsatellite instability (MSI), caused by a deficiency in the DNA mismatch repair system (dMMR), is present in ∼15% of stage II, 10% of stage III, and 5% of stage IV CRC cases.^2^^,^^3^ dMMR/MSI tumors exhibit a high mutational load, leading to the generation of neoantigens that are recognized by the immune system, thereby promoting significant immune cell infiltration within the tumor microenvironment. Over the past decade, dMMR/MSI has been established as a predictive biomarker for the efficacy of immune checkpoint inhibitors (ICIs) across various tumor types.^4^

The advent of ICIs has revolutionized the treatment of dMMR/MSI metastatic CRC, with objective response rates (ORR) ranging from 30% to 70% and 3-year progression-free survival (PFS) rates between 37% and 60%, depending on the population and treatment regimen.^5^, ^6^, ^7^, ^8^, ^9^, ^10^, ^11^ The Keynote-177 trial notably showed that pembrolizumab (P) doubled PFS compared with chemotherapy (median 16.5 months versus 8.2 months).^12^ Similarly, the Checkmate 8HW trial demonstrated that nivolumab combined with ipilimumab (N + I) reduced the risk of progression or death by 79% as compared with chemotherapy and improves PFS when compared with N alone.^13^^,^^14^ These results led to European approval of P and N + I for the first-line treatment of dMMR/MSI metastatic CRC.

More recently, promising findings have emerged for ICIs in the neoadjuvant setting. Since the publication of the NICHE trial in 2020, several studies have confirmed the efficacy of neoadjuvant ICI regimens, reporting pathological complete response (pCR) rates of 43%- 68%.^14^, ^15^, ^16^, ^17^, ^18^, ^19^, ^20^, ^21^ These studies suggest that ICIs could enable curative outcomes in some patients without surgery.^22^ However, these studies were predominantly conducted in highly selected patient populations, typically involving younger patients with Eastern Cooperative Oncology Group (ECOG) performance status (PS) 0 and often in single-center settings. Consequently, the generalizability of these results to routine clinical practice remains uncertain.

To address this gap, this retrospective multicenter cohort study was designed to evaluate real-world prescribing practices for neoadjuvant ICI protocols in nonmetastatic dMMR/MSI colon cancer (CC) in France and to assess their safety and efficacy in a routine clinical setting.

## Methods

### Patients

This study included all consecutive patients with nonmetastatic dMMR/MSI CC who received at least one cycle of neoadjuvant ICIs across six French Association des Gastroentérologues Oncologues (AGEO) centers. dMMR/MSI status was confirmed through immunohistochemistry (loss of MLH1/PMS2 or MSH2/MSH6 protein expression) and molecular testing (unstable microsatellite phenotype) on initial tumor biopsies at each participating center. Patients enrolled in clinical trials and those with metastatic disease were excluded. Toxicity was graded according to National Cancer Institute Common Toxicity Criteria V5.0. This investigator-initiated study was approved by the ethics committee (CERAPHP Assistance Publique des Hopitaux de Paris IRB n°IORG0010044) and conducted in accordance with the European and French Good Clinical Practice guidelines, as well as the Declaration of Helsinki.

### Study design

ICI treatments (either P or N + I) were administered intravenously according to standard clinical practices. The type of ICI regimen and the number of cycles were determined by each investigator after discussion of the patient case in a local multidisciplinary board involving at least a gastrointestinal oncologist, a colorectal surgeon, a radiologist, a pathologist, and a molecular biologist.

Radiological responses were assessed locally using RECIST 1.1 criteria. Objective response rate was defined by the rate of partial and complete response. The type of surgery was determined according to French national guidelines (TNCD.org). Perioperative and post-operative adverse events were recorded retrospectively.

The ypTNM classification was used to assess the pathological stage of the tumor after neoadjuvant treatment. Pathological responses were evaluated by local pathologists based on two criteria: (i) the percentage of residual viable tumor cells and (ii) Mandard tumor regression grade (TRG), ranging from TRG 1 (complete regression) to TRG 5 (no regression). A major pathological response (pMR) was defined as ≤10% residual viable tumor. A complete pathological response (pCR) was defined as 0% residual viable tumor, Mandard TRG 1, and ypT0N0. Patients were classified as good responders if they achieved TRG 1 or 2, and poor responders if they had TRG 3-5.^23^^,^^24^

### Endpoints and statistical analysis

The primary endpoint was pathological response after neoadjuvant ICI treatment, evaluated through TRG, the percentage of residual tumor, and the ypTNM stage. Secondary endpoints included ICI tolerability, perioperative and post-operative adverse events, correlation between radiological and histological responses, recurrence-free survival (RFS), and overall survival (OS). RFS was defined as the time from primary tumor removal (or first immunotherapy cycle if surgery was not performed) to recurrence. OS was defined as the time from diagnosis to death from any cause. Factors predictive of primary resistance to ICIs were investigated. The Fisher’s exact test was used for categorical variables, and the Mann–Whitney *U* test was used for the continuous variables. A *P* value <0.05 was considered statistically significant. Exploratory analyses were performed using the pvalue.io. software (Medistica, Paris, France).^25^

## Results

### Patients

**Figure 1:**
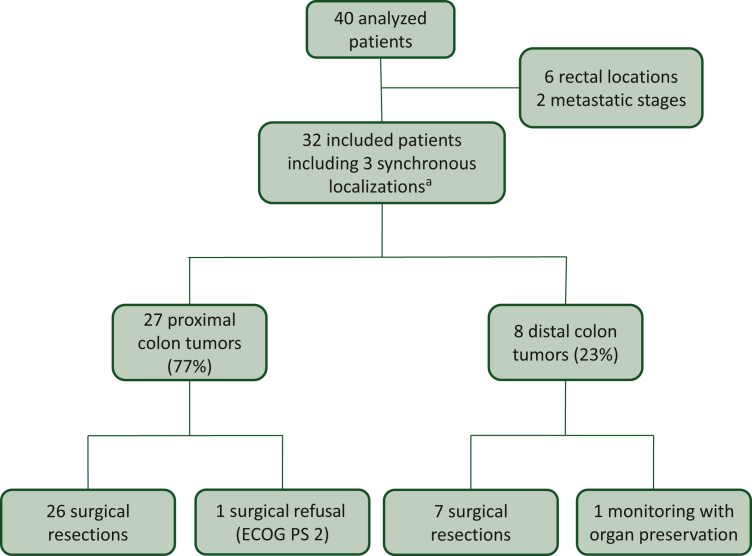
Flow chart. ECOG PS, Eastern Cooperative Oncology Group performance status. ^a^Two ceacum + recto sigmoidal junction, one sigmoid + recto sigmoidal junction.

Between April 2019 and October 2024, 40 patients were reported to study coordinators and 32 patients were included from six French centers (median age 70 years; women 53%). The study population flow chart is presented in Figure 1. Three patients had two synchronous tumor sites, resulting in a total of 35 tumors. Twenty-seven tumors (77%) were located in the proximal colon. Lynch syndrome was identified in 10 patients (31%). The initial characteristics of the patients are presented in Table 1. Baseline characteristics were similar between the P and N + I groups, notably in terms of age, ECOG-PS, tumor location, and tumor computed tomography (CT) staging, except for a nonsignificant trend toward more clinically T4 tumors in the N + I group.

**Table 1:** Patient characteristics

Initial characteristics	Total cohort (*n* = 32)	Pembrolizumab (*n* = 22)	Nivolumab + ipilimumab (*n* = 10)
Age, years^a^	70 (51-76)	70 (50-75)	65 (53-76)
Sex, *n* (%):			
Female	17 (53)	13 (59)	4 (40)
Male	15 (47)	9 (41)	6 (60)
Performance status, *n* (%):			
0	19 (59)	15 (68)	4 (40)
1	11 (34)	6 (27)	5 (50)
2	2 (6)	1 (5)	1 (10)
Body mass index, kg/m^2a^	25.4 (22-28)	24.9 (21-27)	26.3 (22-29)
Primary tumor location, *n* (%):^b^			
Proximal colon	27 (77)	20 (80)	7 (70)
Distal colon	8 (23)	5 (20)	3 (30)
Stage cT:			
T1-3	17 (53)	13 (59)	4 (40)
T4	15 (47)	9 (41)	6 (60)
Stage cN:			
N0	12 (37)	8 (36)	4 (40)
N+	20 (62)	14 (64)	6 (60)
Median tumor size, mm^a^	71.5 (42-79)	66 (41-80)	73 (53-76)
Lynch syndrome, *n* (%)	10 (31)	8 (36)	2 (20)
Median time from diagnosis to start of immunotherapy, weeks^a^	4.4 (3.8-5.6)	4.8 (3.9-5.6)	3.9 (3.6-5.1)
Number of immunotherapy cycles^a^	2 (2-4)	2.5 (2-6)	2 (2-2)
Duration of immunotherapy, weeks^c^	12.3 (7.3-20.8)	15.7 (10.4-26.6)	7.1 (5.6-8.1)

cN, clinical N stage; cT, clinical T stage.^a^

a Median (interquartile range).

b Three patients had two primary colon cancers.

c From first cycle of immune checkpoint inhibitors to surgery.

### Treatment

Ten patients (31%) received the combination nivolumab (3 mg/kg) + ipilimumab (1 mg/kg) for one cycle, followed by one cycle of nivolumab (3 mg/kg) monotherapy 2 weeks later (N + I; NICHE regimen).^14^ Twenty-two patients (69%) received P monotherapy with two main regimens: 400 mg every 6 weeks (*n* = 12 patients) and 200 mg every 3 weeks (*n* = 9 patients). The last patient received three cycles at 200 mg every 3 weeks, followed by three cycles at 400 mg every 6 weeks (Table 1). The median time from diagnosis to the start of immunotherapy was 4.4 weeks [interquartile range (IQR) 3.8-5.6 weeks]. The median number of cycles before surgery was 2 [IQR 2-4 cycles]. The median duration of immunotherapy was 12.3 weeks [IQR 7.3-20.8 weeks]. Eleven patients received adjuvant treatment, which was P for five patients, and chemotherapy for six patients.

### Safety

Toxicity data are detailed in Table 2. Toxicity (any grade) occurred in 12 patients (37%).

**Table 2:** Adverse events

Tolerance and side effects	Total cohort (*n* = 32)	Pembrolizumab (*n* = 22)	Nivolumab + ipilimumab (*n* = 10)
Grade 1-2 toxicities, *n* (%)^a^:	6 (19)	7 (32)	2 (20)
Thyroiditis	2 (6)	1 (4)	1 (10)
Arthritis	2 (6)	2 (9)	1 (10)
Toxidermia	2 (6)	2 (9)	0
Colitis	2 (6)	1 (4)	0
Adrenal insufficiency	1 (3)	1 (4)	0
Grade 3-5 toxicities, *n* (%):	6 (19)	3 (14)	3 (30)
Arthritis^b^	1 (3)	1 (4)	0
Fistulization^b^	2 (6)	0	2 (20)
Bowel obstruction^c^	3 (9)	2 (9)	1 (10)
Death	1 (3)	0	1 (10)

a Three patients developed two different grade 1-2 toxicities.

b Toxicities with treatment discontinuation.

c One patient treated with N + I developed fistulization associated with tumor obstruction.

Grade 1-2 systemic toxicities were observed in six patients: thyroiditis (*n* = 2), arthritis (*n* = 2), dermatitis (*n* = 2), colitis (*n* = 2), and adrenal insufficiency (*n* = 1).

Seven grade ≥3 toxicities were observed in 6 patients (19%), including 3/22 patients treated with P and 3/10 of those treated with N + I. Toxicities leading to treatment discontinuation were reported in three of them: one rheumatoid polyarthritis-like syndrome, and two tumor fistulizations. The other grade ≥3 toxicities were bowel obstruction (associated with tumor fistulization in one patient). Among them, two underwent carcinologic surgery showing pMR and a pCR on the surgical specimen. In addition, one patient who received neoadjuvant N + I died 5 days after surgery due to hypoxic cardiorespiratory arrest in a context of ileus with sepsis and dilated cardiomyopathy. An autopsy was performed revealing a sarcoidosis-like granulomatous myocarditis possibly associated with ICI treatment.

### Operative data and post-operative outcomes

At the data cut-off date of October 2024, one patient refused surgery and another was not resected due to poor general condition (aged 76 years, ECOG-PS 3); both of them were still receiving ICI treatment. In total, 30 out of 32 patients (94%) underwent surgical resection, with a median interval of 6.1 weeks (IQR 2.7-7.9 weeks) between the last ICI dose and surgery.

Severe morbidity (Clavien–Dindo ≥III) occurred in three patients (10%), all within the N + I group. Perioperative data are detailed in Supplementary Table S1, available at https://doi.org/10.1016/j.esmoop.2025.105516.

### Pathological results

**Figure 2:**
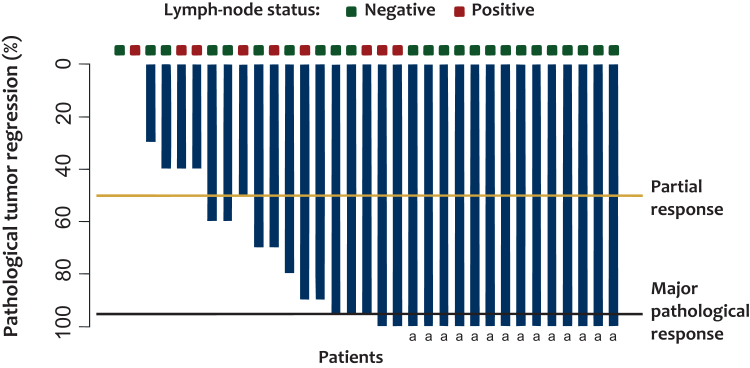
**Pathological response rate.** The waterfall plot shows the percentage of pathological tumor regression per tumor among the 33 tumors that could be evaluated for a pathological response. Boxes above each bar indicate the corresponding pathological lymph-node status. The black horizontal line indicates the threshold for a major pathological response, specified as at least 90% tumor regression. The yellow line indicates the threshold for a partial response, specified as at least a 50% regression. ^a^Patients with a pathological complete response in both the primary tumor and the lymph nodes.

Altogether, 30 patients were resected and 33 tumors were removed. Pathological results are detailed in Figure 2 and Supplementary Table S2, available at https://doi.org/10.1016/j.esmoop.2025.105516. Of the 33 tumors analyzed, 64% achieved a pMR, including 42% with a pCR. pCR rates were 67% after N + I and 33% after P. There was no statistical difference in TRG according to regimen (*P* = 0.22) (Supplementary Figure S1, available at https://doi.org/10.1016/j.esmoop.2025.105516). In the P group, two patients were classified TRG 1 with no residual tumor cells visible on the primary tumor site but had detectable tumor cells in their examined lymph nodes and were thus not classified as pCR.

The three patients operated on with two synchronous tumors showed similar histological responses for the two tumor sites (one was TRG 1 for both tumors and two were TRG 3 for both tumors).

Altogether, 11 tumors (33%) were classified as ‘poor responders’ to ICI: 4 (12%) with TRG 4 and 100% viable tumor cells, and 7 (21%) with TRG 3. The presence of an independent-cells contingent on the surgical specimen was associated with a poor response (TRG 3 or 4, *P* < 0.01) and was observed in 5 of the 11 poor-responding tumors (45%). Of note, the presence of an independent-cells contingent was never detected in the initial biopsy.

### Radiological versus pathological response

After neoadjuvant ICI, five tumors showed a complete radiological response (14%); all of them were classified with a pCR at the pathological examination of their surgical specimen. For patients classified with partial radiological response or stable disease on CT scan, the correlation with pathological outcomes was less clear, as shown in Supplementary Figure S2 and Table S3, available at https://doi.org/10.1016/j.esmoop.2025.105516. For example, 11 pCR tumors were classified as partial radiological response or stable disease.

### Survival

Survival data are detailed in Table 3. With a median follow-up of 18 months, the median RFS was not reached [one locoregional recurrence 13 weeks after ICIs (surgical recusal), one metastatic recurrence 45 weeks after surgery]. With the death of two patients, the median OS was not reached. One patient died 5 days after surgery as mentioned previously and the second patient died 6 months post-operatively due to sepsis without disease recurrence.

**Table 3:** Radiological response and survival data

	Total cohort (*n* = 32) (%)	Pembrolizumab (*n* = 22) (%)	Nivolumab + ipilimumab (*n* = 10) (%)
ORR	24 (70)	18 (75)	6 (60)
Follow-up, weeks^a^	74.5 (64-108)	71.9 (63-89)	113.9 (72-140)
Adjuvant treatment, *n* (%):	10 (31)	8 (36)	2 (20)
Pembrolizumab	4 (12)	4 (18)	0
FOLFOX^b^	5 (16)	3 (14)	2 (20)
CAPOX	1 (3)	1 (4)	0
Recurrence, *n* (%):			
Local	1 (3)	0	1 (10)
Metastatic	1 (3)	1 (4)	0
Deaths, *n* (%):	2 (6)	0	2 (20)

CAPOX, CAPECITABINE + OXALIPLATINE; FOLFOX, LV5FU2 + OXALIPLATINE; ORR, objective response rate (RECIST V1.1).

a Median (interquartile range).

b One patient received adjuvant FOLFOX followed by pembrolizumab for recurrence.

## Discussion

This study is, to our knowledge, the first real-world multicenter cohort to evaluate neoadjuvant immunotherapy in nonmetastatic CC. It investigates the efficacy of P and N + I protocols, which are approved for metastatic disease, in a neoadjuvant setting as previously reported in open-labeled phase II studies. Unlike clinical trials, this cohort included unselected patients, many of whom were older or frail (age >70 years: 50%, ECOG-PS >0: 41%). In this real-world setting, 64% of the 33 tumors histologically analyzed achieved a pMR, including 42% with a pCR. Notably, all complete radiological responses correlated with pCR. However, other radiological RECIST assessment outcomes were not perfectly correlated with pathological response. Grade ≥3 toxicities occurred in 19% of patients, including one suspected treatment-related death.

Published clinical trials show similar pCR rates between monotherapy and combination therapy: 68% with N + I in NICHE-2^14^ and 68% with nivolumab + relatlimab in NICHE-3,^17^ 65% with toripalimabin in the PICC study,^15^ and 79% with P in an MD Anderson study.^16^ In our cohort, the pCR rate was 67% in the N + I group, consistent with these studies, but only 33% in the P group. Similarly, a Chinese observational cohort reported a pCR of 47.6% after a single-agent anti-programmed cell death protein 1 (anti-PD-1) therapy, and the IMHOTEP trial reported a pCR of 54.4% with P in its CRC cohort, which were lower than in previous clinical trials.^19^^,^^26^ These discrepancies underline the importance of comparing results obtained in clinical trials with those obtained in real-world cohorts, and suggest that monotherapy may be less effective than combination therapy. However, our pCR rate was also lower in patients treated with P than those reported in previous studies mentioned above. Of note, the median number of cycles before surgery in our P group was 2.5, similar to the number of cycles in the recent studies IMHOTEP and NICHE 2 and 3, which might indicate a current trend toward shorter treatment duration. This number, however, was lower than the six and eight cycles, respectively, administrated in the PICC and MD Anderson monotherapy trials.^16^^,^^17^ This might explain the different pCR rates between those studies. Recently, early results from the CRC cohort of the IMHOTEP trial suggest an association between the number of neoadjuvant cycles with P and pCR rates.^19^ Further research is needed to optimize neoadjuvant ICI protocols, including the choice between monotherapy and combination therapy together with the optimal duration of treatment.

This study identified a significant association between poor histological response (TRG 3-4) and the presence of signet ring cells (*P* < 0.01). To our knowledge, this association has not been previously reported in this setting. Additionally, the presence of mucin in the surgical specimen was also a factor associated with ICI resistance (*P* < 0.01), as suggested by a case report^27^ and preclinical data.^28^ However, these pathological features can be difficult to identify in diagnostic biopsies and may arise only as a consequence of tumor clone selection during ICI treatment.

Importantly, the rate of grade ≥3 toxicity in our study was higher than in published trials, including one suspected case of treatment-related death. These results underline the need for caution when interpreting efficacy and safety data from clinical trials conducted on selected patients, compared with what may be observed in the context of real-world use. In our work, 19% of patients experienced grade ≥3 toxicity, compared with 4% in NICHE-2 and 3% in the PICC study. Notably, one patient died of granulomatous myocarditis 2.5 months after completing N + I therapy. Although a direct causal link to ICI cannot be confirmed due to the lack of pretreatment cardiac evaluation, toxicity of immunotherapy is possible. Indeed, immuno-induced granulomatosis is a rare but previously described adverse event in the literature.^29^^,^^30^ These results underscore the importance of careful monitoring for immune-related adverse events and considering potential toxicity in therapeutic decision making, especially in the neoadjuvant setting in these patients with a good outcome with surgery alone.

Tumor-related obstruction occurred in 9% of patients, with two showing histological responses (one pCR, one pMR) after surgery. The occurrence of obstruction under ICI could therefore represent an excessively strong immune response. In a recently published European cohort reporting nine cases of obstruction occurring in patients with CC treated with ICIs, only four had pCR. In the same cohort, the rate of obstruction under ICI was only estimated at 1.5%.^31^ In this cohort, common features associated with bowel obstruction, including hepatic flexure location, T4 radiological staging, annular shape, radiological stricturing, and endoscopic obstruction, were described, and should be sought out by clinicians to alert patients to this risk and to the need for prompt consultation in the event of obstructive symptoms. Larger real-world studies are necessary to better understand this phenomenon and anticipate it, for example, by considering a preventive diverting stoma before initiating immunotherapy.

This study has several limitations that should be acknowledged. Firstly, its retrospective design and the small sample size, consisting of 32 patients from six centers, limit the generalizability of the findings. Secondly, the heterogeneity in follow-up durations hinders a comprehensive assessment of recurrence risk. Thirdly, there was an absence of central review of baseline CT scans, with some patients having small tumors that may not have needed such preoperative treatments. Finally, the lack of standardized neoadjuvant protocols introduces variability in treatment regimens and duration, with P accounting for 69% of the prescriptions.

Despite these limitations, this study generates the first real-world evidence on preoperative ICI in MSI/dMMR nonmetastatic CC patients and valuable insights into real-world prescribing practices and patient outcomes. It shows less pMR and pCR than reported previously and more severe toxicities, highlighting the need for more standardized patient selection criteria and preoperative treatment regimens to optimize tolerability and efficacy of this new treatment possibility.
