# Supplementary material for: Neoadjuvant immunotherapy for nonmetastatic dMMR/MSI colon cancer: a real-world retrospective AGEO study
Source: ESMO Open. 2025 Jul 31;10(8):105516. doi: 10.1016/j.esmoop.2025.105516 (PMC12337657; doi:10.1016/j.esmoop.2025.105516)
Supplement: Supplementary Data [file mmc1.docx]

**Pre-Op IT : real-word data of neoadjuvant immunotherapy for non-metastatic dMMR/MSI colon cancer.**

Pre-Op IT is the first multicenter retrospective real-world study of neoadjuvant immunotherapy for non-metastatic dMMR/MSI colon cancer. It showed lower pMR and pCR rates than those reported in phase II studies (respectively 64% and 42%) and more severe toxicities (19%), including one toxic death.

These results highlight the need for more standardized patient selection criteria and preoperative treatment regimens to optimize tolerability and efficacy of this new treatment possibility.

Twitter : @GillesManceau @AnnaliceGandini
