# Supplementary material for: Neoadjuvant immunotherapy for nonmetastatic dMMR/MSI colon cancer: a real-world retrospective AGEO study
Source: ESMO Open. 2025 Jul 31;10(8):105516. doi: 10.1016/j.esmoop.2025.105516 (PMC12337657; doi:10.1016/j.esmoop.2025.105516)
Supplement: Supplementary Figures [file mmc2.docx]

**Supplementary data:**

**Figure S1: Correlation between radiological and histological response**


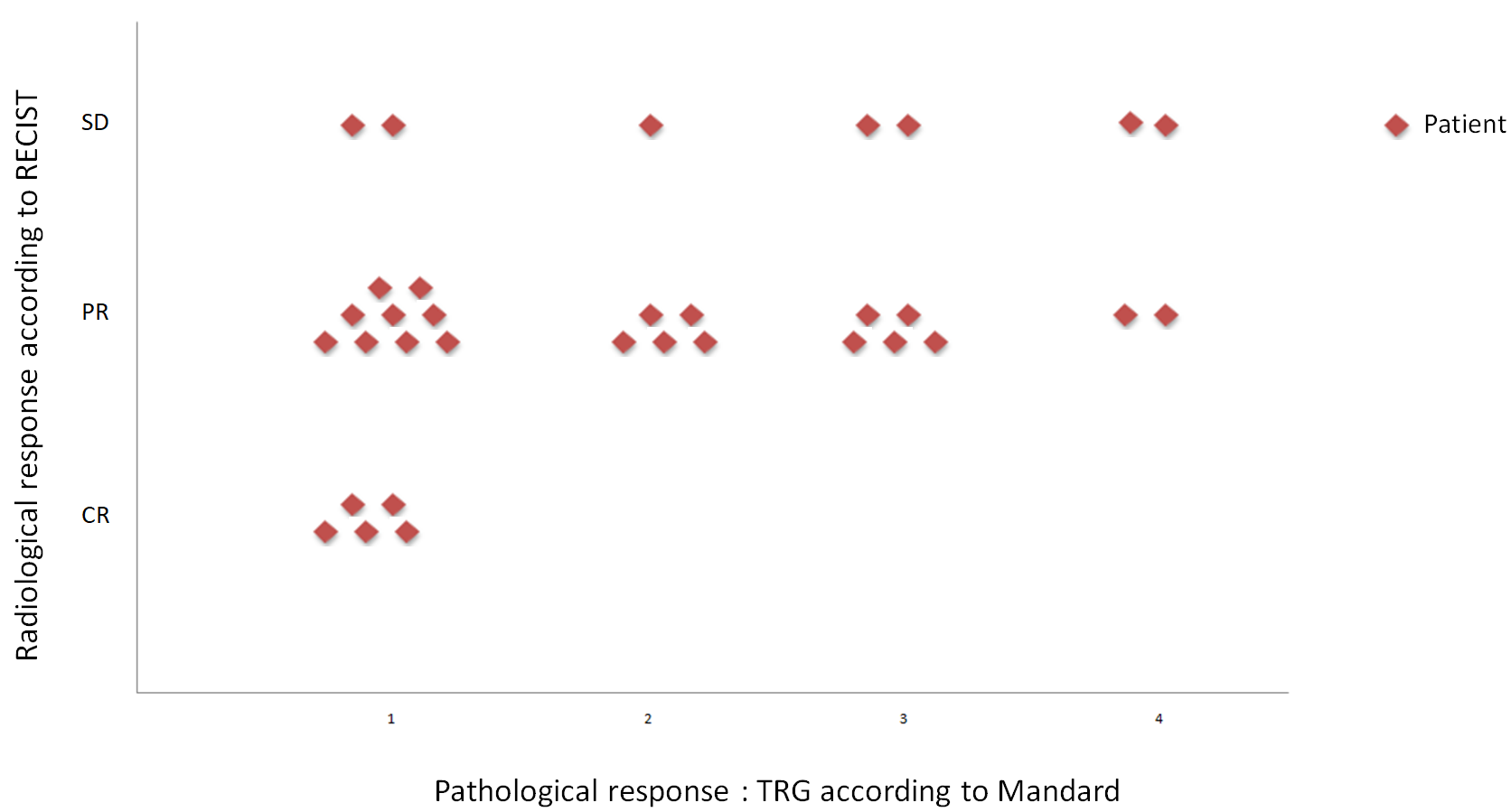


**Figure S2: Pathological response (TRG) according to ICI protocole**

**
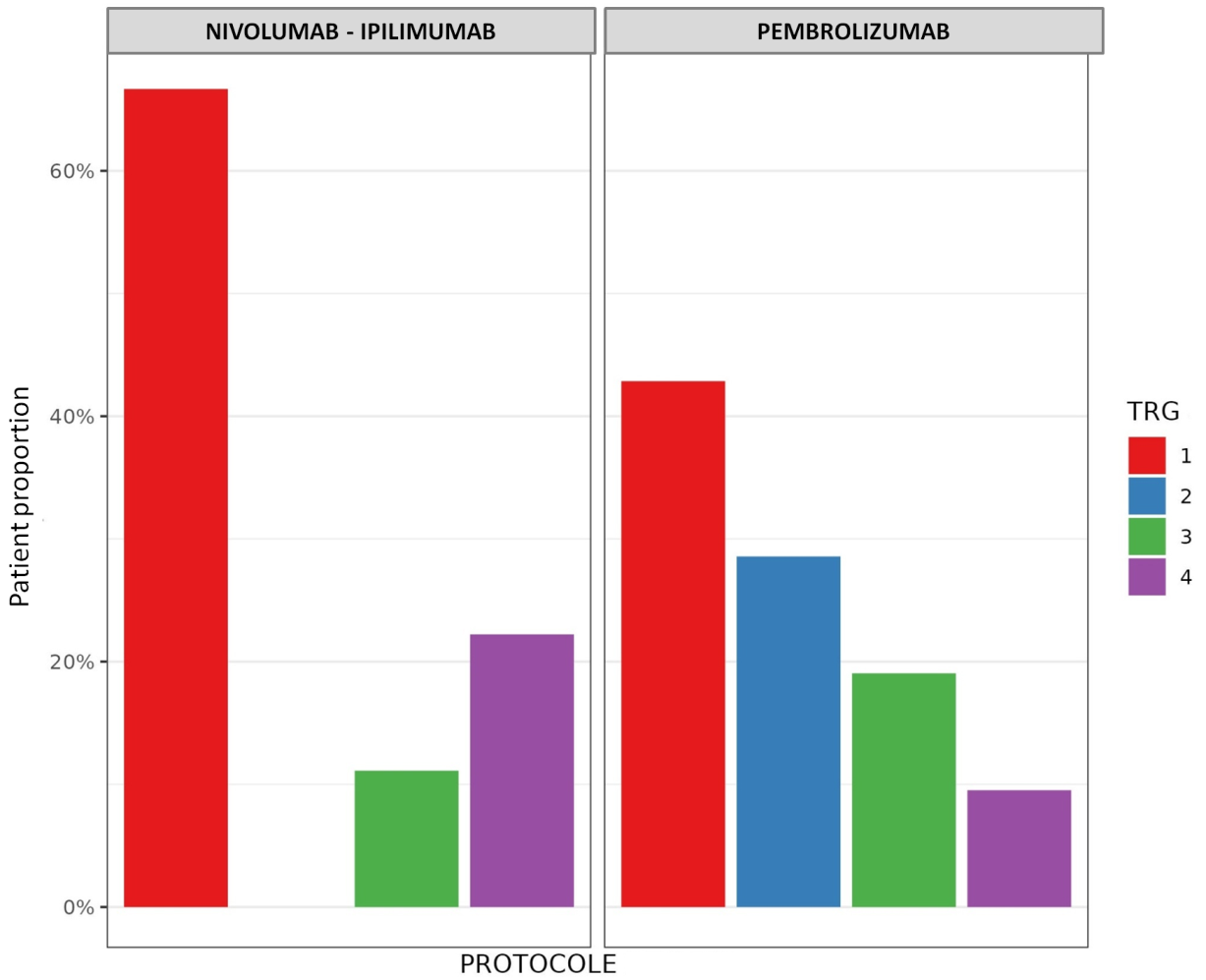
**
