# Supplementary material for: Neoadjuvant immunotherapy for nonmetastatic dMMR/MSI colon cancer: a real-world retrospective AGEO study
Source: ESMO Open. 2025 Jul 31;10(8):105516. doi: 10.1016/j.esmoop.2025.105516 (PMC12337657; doi:10.1016/j.esmoop.2025.105516)
Supplement: Supplementary Tables [file mmc3.docx]

**Supplementary data:**

**Table S1: Operative data and postoperative results**

| **Operating data** | **Total cohort**  **(n=30) (%)** | **pembrolizumab**  **(n=21) (%)** | **nivolumab + ipilimumab**  **(n=9) (%)** | **p** |
| --- | --- | --- | --- | --- |
| **Surgical approach**, n (%): |  |  |  | 0.72 |
| Laparotomy | 12 (40) | 8 (38) | 4 (40) |  |
| Laparoscopy | 15 (50) | 10 (48) | 5 (50) |  |
| Robotic-assisted  Conversion | 3 (10)  3 (10) | 3 (14)  3 (14) | 0  0 |  |
|  |  |  |  | 0.53 |
| **Type of colectomy**, n (%): |  |  | 0  7 (78)  0  1 (11)  0  1 (1)  5 (50) | 0.68 |
| Ileocaecal resection  Right hemicolectomy  Left colectomy  Subtotal colectomy  Ileocecal resection and  left colectomy  Other  Resection extended to  a neighbouring organ, n (%) | 2 (6)  22 (73)  1 (3)  1 (3)  2 (7)  2(7)  9 (30) | 2 (9)  15 (71)  1 (5)  0  2 (9)  1 (5)  4 (20) |  |  |
|  |  |  |  |  |
|  |  |  |  |  |
|  |  |  |  |  |
|  |  |  |  |  |
|  |  |  |  |  |
|  |  |  |  | 0.082 |
| **Stoma,** n (%) | 5 (17) | 4 (19) | 1 (11) | 1  0.44 |
| **Operating time**, min* | 211 [164-248] | 233 [162-274] | 201 [184-210] |  |
| **Postoperative complication**, n (%) | 10 (33) | 5 (24) | 5 (55) | 0.2  0.089 |
| **Clavien-Dindo complication**, n (%): |  |  |  |  |
| Grade I | 3 (10) | 2 (9) | 1 (11) |  |
| Grade II | 4 (13) | 3 (14) | 1 (11) |  |
| Grade III | 1 (3) | 0 | 1 (11) |  |
| Grade IV | 1 (3) | 0 | 1 (11) |  |
| Grade V | 1 (3) | 0 | 1 (11) |  |
| **Anastomotic leakage,** n (%) | 2 (7) | 1 (5) | 1 (11) | 0.52 |
| **Unplanned reintervention,** n (%) ** | 2 (7) | 0 | 2 (22) | 0.083 |
| **Length of hospital stay**, days * | 7.5 [6-11] | 7 [6-9] | 8 [6-13] | 0.41 |

* median [IQR1-3] ; ** due to anastomotic leakage

**Table S2: Histological findings**

| **Histological findings on resected tumors** | **Total cohort**  **(n=33) (%)** | **pembrolizumab**  **(n=24) (%)** | **nivolumab + ipimilumab**  **(n=9) (%)** | **p** |
| --- | --- | --- | --- | --- |
| **Tumor size**, mm *^,^ | 39.5 [23-45] | 40 [25-45] | 30 [15-45] | 0.55 |
| **Length of resection specimen**, cm *^,^ | 33 [25-34] | 30 [22-34] | 34 [32-43] | 0.052 |
| **Stage ypT**, n (%): |  |  |  | 0.61 |
| ypT0 | 16 (48) | 10 (42) | 6 (66) |  |
| ypT1 | 5 (15) | 4 (17) | 1 (11) |  |
| ypT2 | 3 (9) | 3 (12) | 0 |  |
| ypT3 | 4 (12) | 4 (17) | 0 |  |
| ypT4a | 2 (6) | 1 (4) | 1 (11) |  |
| ypT4b | 3 (9) | 2 (8) | 1 (11) |  |
| **Stage ypN,** n (%): |  |  |  | 0.15 |
| ypN0 | 25 (76) | 18 (75) | 7 (78) |  |
| ypN1a | 4 (12) | 5 (21) | 0 |  |
| ypN1b | 2 (6) | 1 (4) | 1 (11) |  |
| ypN1c | 0 | 0 | 0 |  |
| ypN2a | 1 (3) | 0 | 1 (11) |  |
| ypN2b | 0 | 0 | 0 |  |
| **Number of lymphaticnodesexamnied** *^,^ | 24.5 [20-32] | 26 [22-36] | 22 [19-22] | 0.069 |
| **Stage ypTNM**, n (%) **: |  |  |  |  |
| pCR (ypT0N0M0) | 14 (42) | 8(33) | 6 (67) |  |
| Stage I | 7 (21) | 6 (25) | 1 (11) |  |
| Stage II | 3 (9) | 3 (12) | 0 |  |
| Stage III | 9 (27) | 7 (29) | 2 (22) |  |
| **Tumour TRG**, n (%) **: |  |  |  | 0.22 |
| 1 | 16 (48) | 10 (42) | 6 (67) |  |
| 2 | 6 (18) | 6 (25) | 0 |  |
| 3 | 7 (21) | 6 (25) | 1 (11) |  |
| 4 | 4 (12) | 2 (8) | 2 (22) |  |
| **Percentage of residual tumor cells**:** |  |  |  | 0.73 |
| 0 | 16 (48) | 10 (42) | 6 (67) |  |
| < 10 | 5 (15) | 5 (21) | 0 |  |
| 10 to 50 | 6 (18) | 6 (25) | 0 |  |
| 50 to 100 | 2 (6) | 1 (4) | 1 (11) |  |
| 100 | 4 (12) | 2 (8) | 2 (22) |  |
| **Vascular invasion**, n (%)  **Lymphatic invasion**, n (%)  **Perineural invasion**, n (%)  **Mucinousresponse**, n (%)  **Fibrousresponse**, n (%)  **Gigantocellularepithelioidgranuloma**, n (%)  **Independent/signet ring cells**, n (%)  **Independent cells amongTRG 3, n (%)**  **Independent cells among TRG 4, n (%)** | 0  4 (12)  1 (3)  20 (60)  21 (64)  5 (15)  5 (15)  4 (57)  1 (25) | 2 (8)  1 (4)  14 (58)  15 (62)  4 (17)  4 (17)  3(50)  1 (50) | 2 (22)  0  6 (67)  6 (67)  1 (11)  1 (11)  1 (100)  0 | 0.21  1  0.67  0.85  1  1 |

pCR: complete pathological response; * median [IQR]; ** Two patients in the P group were TRG1 with 0 residual tumor cell in the primary tumor site but had residual tumor cells in theirlymphnodes

**Table S3: Radiological response**

| **Radiological response after ICI** | **Total cohort**  **(n=35) (%)** | **pembrolizumab**  **(n=25) (%)** | **nivolumab**  **+ipilimumab**  **(n=10) (%)** | **p** |
| --- | --- | --- | --- | --- |
| **T Stage,** n (%): * |  |  |  | 0.4 |
| T0 | 5 (14) | 5 (20) | 0 |  |
| T1 | 4 (11) | 4 (16) | 0 |  |
| T2 | 9 (26) | 6 (24) | 3 (30) |  |
| T3 | 2 (6) | 1 (4) | 1 (10) |  |
| T4a | 7 (20) | 4 (16) | 3 (30) |  |
| T4b | 8 (23) | 5 (20) | 3 (30) |  |
| **N stage**, n (%): * |  |  |  | 1 |
| N0 | 21 (60) | 15 (60) | 6 (60) |  |
| N1 | 8 (23) | 5 (20) | 3 (30) |  |
| N2 | 6 (17) | 5 (18) | 1 (10) |  |
| **TNM stage**, n (%): * |  |  |  | 0.14 |
| CR | 5 (14) | 5 (20) | 0 |  |
| Stage I | 7 (20) | 6 (24) | 1 (10) |  |
| Stage II | 9 (26) | 4 (16) | 5 (50) |  |
| Stage III | 14 (40) | 10 (4) | 4 (40) |  |
| **RECIST,** n (%): * |  |  |  | 0.24 |
| CR | 5 (14) | 5 (20) | 0 |  |
| PR | 22 (63) | 16 (64) | 6 (60) |  |
| SD | 8 (23) | 4 (16) | 4 (40) |  |
| **Type of radiological response,** n (%): ** |  |  |  |  |
| Fibrotic | 10 (43) | 6 (37) | 4 (57) | 0.39 |
| Mucinous | 11 (48) | 7 (44) | 4 (57) | 0.67 |
| Necrotic | 4 (17) | 3 (19) | 1 (14) | 1 |

CR: complete response; PR: partial response, SD: stable disease *three patients had synchronous tumors; ** in the subgroup of patients who had centralized rereading, i.e. 20 patients, 23 tumours (different types of responses could co-exist in one tumour).
